# Comparison of Oxidative Stress/DNA Damage in Semen and Blood of Fertile and Infertile Men

**DOI:** 10.1371/journal.pone.0068490

**Published:** 2013-07-12

**Authors:** Jolanta Guz, Daniel Gackowski, Marek Foksinski, Rafal Rozalski, Ewelina Zarakowska, Agnieszka Siomek, Anna Szpila, Marcin Kotzbach, Roman Kotzbach, Ryszard Olinski

**Affiliations:** 1 Department of Clinical Biochemistry, Collegium Medicum, Nicolaus Copernicus University, Bydgoszcz, Poland; 2 Gynecology practice, Specialist medical practice, Bydgoszcz, Poland; 3 Department of Obstetric Nursing, Collegium Medicum, Nicolaus Copernicus University, Bydgoszcz, Poland; University of Hawaii at Manoa, John A. Burns School of Medicine, United States of America

## Abstract

Abnormal spermatozoa frequently display typical features of oxidative stress, i.e. excessive level of reactive oxygen species (ROS) and depleted antioxidant capacity. Moreover, it has been found that a high level of oxidatively damaged DNA is associated with abnormal spermatozoa and male infertility. Therefore, the aim of our study was the comparison of oxidative stress/DNA damage in semen and blood of fertile and infertile men. The broad range of parameters which describe oxidative stress and oxidatively damaged DNA and repair were analyzed in the blood plasma and seminal plasma of groups of fertile and infertile subjects. These parameters include: (i) 8-oxo-7,8-dihydro-2′-deoxyguanosine (8-oxodG) and 8-oxo-7,8-dihydroguanine (8-oxoGua) levels in urine; (ii) 8-oxodG level in DNA isolated from leukocytes and spermatozoa; (iii) antioxidant vitamins (A, C and E) and uric acid. Urinary excretion of 8-oxodG and 8-oxoGua and the level of oxidatively damaged DNA in leukocytes as well as the level of antioxidant vitamins were analyzed using HPLC and HPLC/GC/MS methods.

The results of our study demonstrate that 8-oxodG level significantly correlated with every parameter which describe sperm quality: sperm count, motility and morphology. Moreover, the data indicate a higher level of 8-oxodG in sperm DNA compared with DNA of surrogate tissue (leukocytes) in infertile men as well as in healthy control group. For the whole study population the median values of 8-oxodG/10^6^ dG were respectively 7.85 and 5.87 (p = 0.000000002). Since 8-oxodG level in sperm DNA is inversely correlated with urinary excretion rate of 8-oxoGua, which is the product of OGG1 activity, we hypothesize that integrity of spermatozoa DNA may be highly dependent on OGG1 activity. No relationship between the whole body oxidative stress and that of sperm plasma was found, which suggests that the redox status of semen may be rather independent on this characteristic for other tissues.

## Introduction

One of the major factors which define fertility is the quality of spermatozoa and it has been recently estimated that one in twenty men exhibits some form of defect in sperm quality, which in turn may be involved in male factor infertility which is responsible for half of all infertility cases [1].

There is considerable evidence that oxidative stress plays a major role in the etiology of male infertility. Thus, abnormal spermatozoa frequently display typical features of oxidative stress i.e. excessive level of reactive oxygen species (ROS) and depleted antioxidant capacity [2]–[4]. Moreover, it has been found that a high level of oxidatively damaged DNA is associated with abnormal spermatozoa and male infertility [5].

Despite significant progress in the last decade regarding a possible role of oxidative stress in the etiology of defective sperm function and male infertility some important issues, outlined below, have not yet been addressed: (i) whether oxidative stress in patients with abnormal spermatozoa is restricted to semen only, or whether the whole organism is oxidatively stressed as well; (ii) whether there is any association between oxidatively damaged DNA in sperm and that observed in surrogate tissue (leukocytes) which represents other cells in the body.

When investigating a possible link between oxidative stress and abnormal spermatozoa it is important to apply an appropriate biomarker of oxidative stress. The most popular way of exploring oxidative stress includes measures of oxidative DNA damage which can be assessed by determination of 8-oxodG level in cellular DNA. It is also believed that lymphocytes are surrogate cells, which should inform about oxidative stress - measured as a certain level of 8-oxodG - in other tissues [6], [7]. In addition, the whole body burden of oxidative stress may be assessed by the determination of urinary excretion of oxidatively modified bases/nucleosides [8], [9]. Therefore, in the present study, for the first time the broad range of parameters which describe oxidative stress and oxidatively damaged DNA and repair were analyzed in the groups of fertile and infertile subjects. These parameters include: (i) 8-oxodG level in DNA isolated from leukocytes and spermatozoa; (ii) 8-oxodG and 8-oxoGua levels in urine; (iii) antioxidant vitamins (A, C and E) and uric acid. Low molecular weight antioxidants are regarded as effective free radical scavengers, therefore they can protect biomolecules such as DNA either directly or indirectly. Therefore, in addition to the aforementioned analyses of oxidative DNA damage biomarkers, the concentration of antioxidant vitamins A, C, E and uric acid was determined in the blood serum and seminal plasma (ascorbic and uric acid) of the subjects.

## Materials and Methods

### Subjects

Ethics statement: The study was approved by the bioethics committee, Collegium Medicum in Bydgoszcz, Nicolaus Copernicus University in Torun, Poland (No KB/396/2009). From all participants involved in this study we have obtained informed written consent. Information regarding body weight, height, smoking, eating habits and age was obtained from a questionnaire.

The males included in this study were recruited from healthy volunteers and patients attending the andrology outpatient clinic. The patients group included 91 infertile men with abnormal semen parameters. The group was further divided into the following subgroups: asthenozoospermia (n = 47; progressive motility <32%), oligozoospermia (n = 6; sperm concentration <15×10^6^/ml), oligoasthenozoospermia (n = 31; sperm concentration <15×10^6^/ml and progressive motility <32%), cryptozoospermia (n = 7; spermatozoa absent from fresh preparations but observed in a centrifuged pellet). The control group consisted of 32 healthy donors who had normal semen characteristics (normozoospermia). Among the control patients 19 men had fathered at least one child. The remaining 13 men had no history of infertility and their sperm quality fulfilled all the criteria for normozoospermia. Five men in the control group and 25 of the patients group were reported to be cigarette smokers. The female partners of infertile men had been evaluated by a gynecologist and were diagnosed as potentially fertile. The characteristics of patients and their semen quality are presented in Table 1.

**Table 1:** Characteristic of subjects’ age and semen quality parameters of study groups.

Parameter	Control group/normozoospermian = 32	Patients				
		All n = 91	Asthenozoospermian = 47	Oligoasthenozoospermian = 31	Oligozoospermian = 6	Cryptozoospermian = 7
**Age**	33.0 (28.0–35.5)	33.0(28.0–36.0)	31.0 (28.0–36.0)	32.0 (29.0–35.0)	33.0 (29.0–36.0)	35.0 (28.0–38.0)
**Sperm concentration** **(10^6^/ml)**	37.09 (23.99–63.85)	15.94(8.02–41.85)	40.00 (21.12–61.70)	7.85 (3.71–11.85)	8.38 (8.20–11.02)	<1.00
**Total sperm number** **(10^6^/ejaculate)**	113.90 (74.59–181.40)	42.25(16.72–118.45)	110.36 (58.5–188.80)	16.92 (9.90–31.40)	18.99 (16.53–32.80)	
**Progressive motility** **(%)**	41.0 (35.5–49.5)	10.0(3.0–19.0)	11.0 (5.0–17.0)	7.0 (0.0–11.0)	51.0 (36.0–57.0)	
**Total motility (%)**	60.0 (52.5–64.5)	44.0(29.0–58.0)	44.0 (30.0–59.0)	35.0 (22.0–51.0)	60.0 (58.0–70.0)	
**Sperm morphology/** **normal forms (%)**	20.5 (14.0–30.0)	18.0(12.0–22.0)	19.0 (13.0–22.0)	16.0 (12.0–23.0)	20.5 (17.5–24.5)	
**Round cells** **concentration (10^6^/ml)**	0.56 (0.26–1.10)	0.42(0.16–0.80)	0.54 (0.29–0.97)	0.15 (0.08–0.66)	0.49 (0.40–0.92)	

Values are expressed as median and interquartile range.

### Samples

Semen, urine and venous blood samples were obtained from 123 men (median age 33 years). Semen samples were obtained by masturbation after at least 3 days of ejaculatory abstinence. After semen liquefaction, analysis of semen parameters (i.e. volume, sperm concentration, motility and morphology) was performed according to WHO guidelines from 2010 [10]. Sperm morphology was evaluated on stained semen smears by light microscopy. This was followed by sperm separation. The semen samples were centrifuged using Percoll gradients (80% and 40% lyers). After centrifugation, samples were aspirated from the bottom and interface. The resulting sperm pellets were washed three times with phosphate buffered saline (PBS). The blood was carefully applied on top of Histopaque 1119 solution (Sigma, St. Louis, MO) and leukocytes were isolated by centrifugation according to the procedure laid down by the manufacturer.

### DNA Isolation

The pellet of the cells (leukocytes or spermatozoa) was dispersed by vortexing in ice-cold buffer (10 mM Tris-HCl, 5 mM Na_2_EDTA, 0.15 mM deferoxamine mesylate, pH 8.0). Solution of SDS (to the final concentration of 0.5%), and RNase A and T1 (final concentration 5 µg/ml and 5.5 Kunitz units/ml, respectively) were added, and the mixture gently vortexed. After incubation for 30 min. at 37°C, proteinase K was added (to the final concentration 1 mg/ml). In the case of sperm cells dithiothreitol was added to the final concentration of 10 mM. Then, the mixture was gently vortexed and incubated at 37°C for 1 h. The mixture was cooled to 4°C and transferred to a centrifuge tube with Tris-buffered phenol pH 8.0/chloroform/isoamyl alcohol (25∶24:1 (v/v/v)) and vortexed vigorously. After extraction aqueous phase was treated with chloroform/isoamyl alcohol mixture (24∶1 (v/v)). Supernatant containing DNA was treated with 2 volumes of cold absolute ethanol in order to precipitate high molecular weight DNA. The precipitate was removed with a plastic spatula, washed with 70% ethanol, dried and dissolved in 0.1% (v/v) CH_3_COOH containing 0.2 mM ZnCl_2_.

### 8-oxodG Determination in DNA Isolates

Dissolved DNA samples (100 µl) were mixed with 1 U of nuclease P1. Samples were incubated at 37°C for 1 h. Thereafter 20 µl 1% (v/v) NH_4_OH and 1.3 U of alkaline phosphatase were added to each sample following incubation at 37°C for 1 h. All DNA hydrolysates were ultrafiltered using cut-off 5000 Da filter units. 8-OxodG and dG in hydrolysates were determined using HPLC. The HPLC system consisted of Dionex GP40 pump, Waters 717 autosampler, 250×4.6 mm LC18S column (5 µm grain) equipped with 20 mm precolumn and with two detectors working in series: UV-VIS (Waters 2487) and Coulochem II 5200A electrochemical detector (ESA, Inc., Chelmsford, MA, USA). DNA hydrolysates were chromatographed isocratically using 25 mM sodium acetate, 12.5 mM citrate, pH 5.0/methanol (88∶12 (v/v)). Detection of dG was performed at 254 nm. 8-OxodG was determined by the electrochemical detector: guard cell +750 mV, detector 1: +130 mV (as a screening electrode), detector 2: +450 mV (as a measuring electrode set to sensitivity of 50 nA/V). Acquisition and quantitative analysis of chromatograms was carried out using Chromeleon 6 software (Dionex Corporation). The amount of 8-oxodG in DNA was calculated as the number of 8-oxodG molecules per 10^6^ unmodified dG molecules.

### Determination of Vitamins A, E, C and Uric Acid Concentration by HPLC

Quantification of vitamin E (α-tocopherol), vitamin A (retinol), vitamin C (ascorbic acid) and uric acid by HPLC technique was described previously [11].

### Determination of 8-oxoGua and 8-oxodG in Urine

Urine sample preparation, HPLC purification and GC/MS analysis were conducted as described earlier [11], [12].

### Statistical Analysis

For the statistical analysis, the STATISTICA (data analysis software system, version 10.0) from StatSoft, Inc. was used. For normal distribution, variables were analyzed by the Kolmogorov-Smirnov test. The statistical significance was assessed using nonparametric (Wilcoxon and Mann–Whitney U) tests and presented as median values and interquartile range. The correlations were assessed using the Spearman’s correlation analysis. Statistical significance was considered at p<0.05.

## Results

Compared with healthy donors, infertile patients had significantly lower sperm concentration (p = 0.00005), total sperm number (p = 0.0002) and reduced progressive and total motility (p = 0.000000000003 and p = 0.00002, respectively). Patients also had worse sperm morphology than healthy donors (p = 0.03). Table 1 summarizes the semen quality of the study groups.

**Figure 1:**
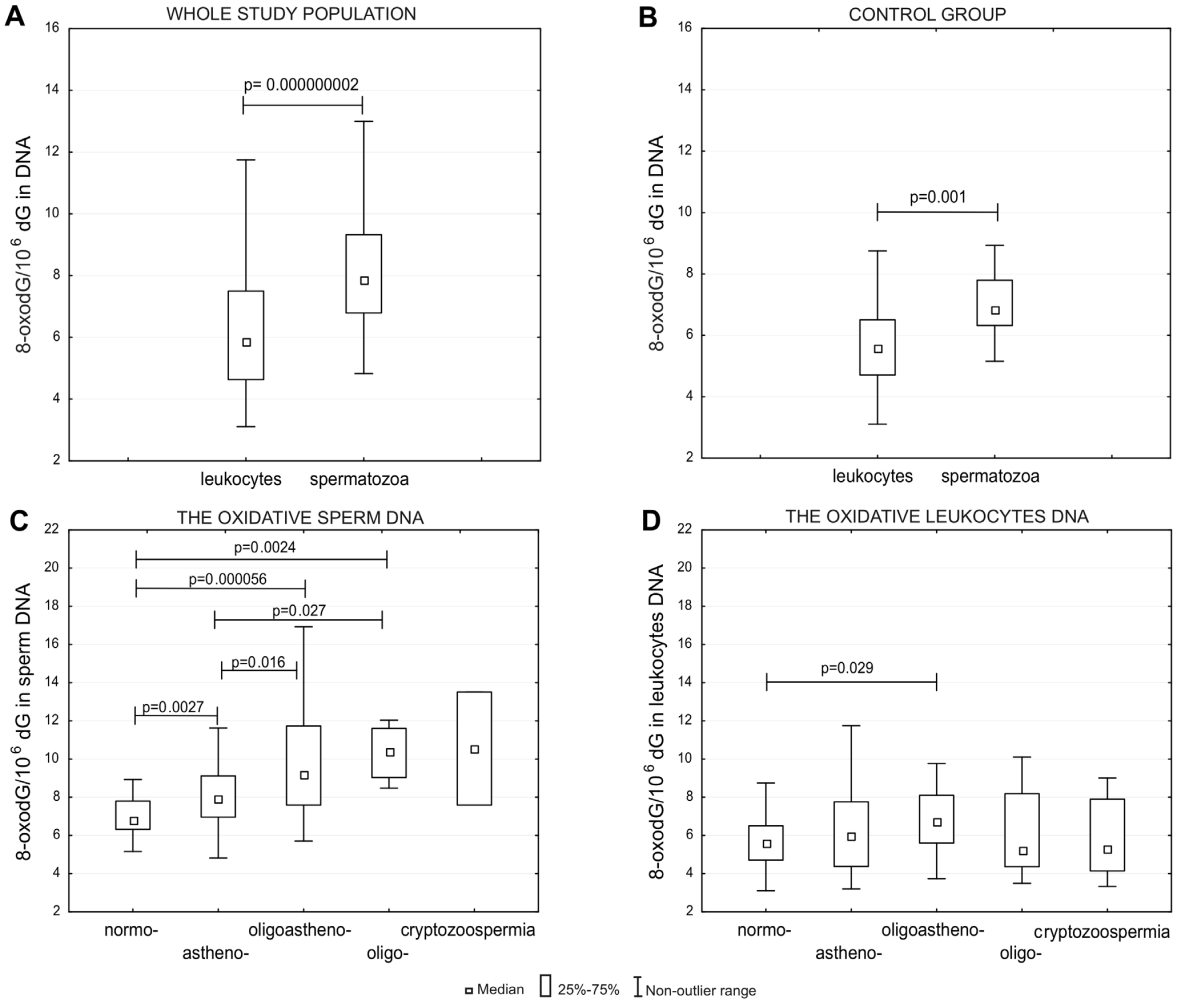
Levels of 8-oxodG in spermatozoa and leukocytes. A, Levels of 8-oxodG in leukocytes DNA compared with sperm DNA in samples from all studied subjects. B, Levels of 8-oxodG in leukocytes DNA and sperm DNA in control group. C, Levels of 8-oxodG in sperm DNA in different studied groups. D, Levels of 8-oxodG in leukocytes DNA in different studied groups.

8-OxodG level was measured in DNA isolated from the spermatozoa and leukocytes. For the whole study population (fertile and infertile subjects), the median values of 8-oxodG/10^6^ dG were respectively 7.85 (4.63–7.50) and 5.87 (6.79–9.33). This difference is highly statistically significant (p = 0.000000002) (Figure 1A). Similarly, the level of 8-oxodG in sperm DNA was significantly higher compared with DNA isolated from leukocytes in control group with normal sperm parameters (Figure 1B) as well as in every study subgroup with sperm dysfunction. No significant correlation was observed between levels of 8-oxodG in leukocytes DNA and sperm DNA (data not shown).

Determination of 8-oxodG in sperm DNA of different study groups revealed significantly higher level of the modification in the whole group of patients (median value 8.43 of 8-oxodG/10^6^ dG) in comparison with healthy control group (median value 6.83 of 8-oxodG/10^6^ dG). Detailed comparison showed the following significant differences: normozoospermia versus: i/asthenozoospermia (p = 0.0027); ii/oligozoospermia (p = 0.0024); iii/oligoasthenozoospermia (p = 0.000056), as well as between asthenozoospermia versus: i/oligoasthenozoospermia (p = 0.016), ii/oligozoospermia (p = 0.027) (Figure 1C). Concerning the analyses of 8-oxodG level in leukocytes DNA the only difference was found in subjects with oligoasthenozoospermia compared to control group (p = 0.029) (Figure 1D). The comparative levels of 8-oxodG in DNA in all studied groups are summarized in Table 2.

**Table 2:** Comparison of the analytical parameters among the study groups.

Parameter	Control group/normozoospermia	Patients				
		All	Asthenozoospermia	Oligoasthenozoospermia	Oligozoospermia	Cryptozoospermia
**8-oxodG/10^6^dG in** **leukocytes DNA**	5.58(4.71**–**6.50)n = 28	6.02(4.70**–**7.88)n = 88	5.98(4.37**–**7.76)n = 46	6.74(5.60–8.10)^a^n = 30	5.26(4.36–8.18)n = 5	5.30(4.14**–**7.90)n = 7
**8-oxodG/10^6^dG** **in sperm DNA**	6.83(6.32–7.80)^1^n = 31	8.43(7.17**–**10.15)a,1n = 77	7.90(6.96**–**9.11)a,1n = 45	9.21(7.59**–**11.73)a,b,1n = 26	10.38(9.04**–**11.60)a,bn = 4	10.54(7.59**–**13.5)n = 2
**Urinary 8-oxoGua** **excretion (nmol/mmol** **creatinine)**	8.20(5.61–12.00)n = 31	6.76(4.67–9.26)n = 87	7.10(4.69–9.26)n = 46	5.93(5.51–8.57)n = 29	6.77(4.25–7.08)n = 5	6.86(4.01–8.60)n = 7
**Urinary 8-oxodG** **excretion (nmol/mmol** **creatinine)**	1.62(1.28–2.11)n = 29	1.48(1.17–1.78)n = 87	1.45(1.14–1.76)n = 46	1.52(1.20–1.77)n = 29	1.36(1.22–2.60)n = 5	1.59(1.14–2.16)n = 7
**Blood plasma ascorbic** **acid (µmol/l)**	45.20(32.61–68.69)^2^n = 32	43.16(31.59–56.68)^2^n = 90	41.97(32.36–58.55)^2^n = 47	43.77(31.56–56.68)^2^n = 31	36.69(23.97–48.95)n = 5	51.28(36.55–65.22)^2^n = 7
**Blood plasma uric acid** **(µmol/l)**	381.14(338.04–432.93)n = 32	372.4(339.97–417.82)n = 90	380.50(339.97–422.70)n = 47	373.80(346.99–415.07)n = 31	357.41(331.80–445.50)n = 5	351.38(318.68–413.10)n = 7
**Blood plasma retinol** **(µmol/l)**	2.19(1.96–2.56)n = 32	2.20(1.92–2.68)n = 90	2.18(1.92–2.67)n = 47	2.43(1.83–2.77)n = 31	2.14(1.97–2.34)n = 5	2.07(2.02-2.21)n = 7
**Blood plasma** **α-tocopherol (µmol/l)**	29.50(25.32–37.60)n = 32	29.24(23.96–34.33)n = 90	28.85(24.61–35.33)n = 47	29.22(23.96–32.25)n = 31	28.65(22.66–30.65)n = 5	34.33(23.86–42.26)n = 7
**Seminal plasma** **ascorbic acid (µmol/l)**	139.57(109.66–399.32)n = 32	181.48(103.82–284.7)n = 90	203(125.73–312.56)n = 47	162.30(93.30–236.50)n = 31	111.94(82.48–141.20)n = 5	235.20(129.44–318.70)n = 7
**Seminal plasma** **uric acid (µmol/l)**	325.40(288.25–402.47)n = 32	336.79(293.7–434.03)n = 90	352.28(304.40–433.83)n = 47	332.49(290.91–443.86)n = 31	248.08(193.48–293.70)n = 5	375.23(276.52–485.50)n = 7

Values are expressed as median and interquartile range. Statistically significant differences (Mann-Whitney U test, p<0.05):

a vs. control group,

b vs. asthenozoospermic patients.

Statistically significant differences (Wilcoxon test, p<0.05):

1 vs. leukocytes,

2 vs. seminal plasma ascorbic acid.

**Figure 2:**
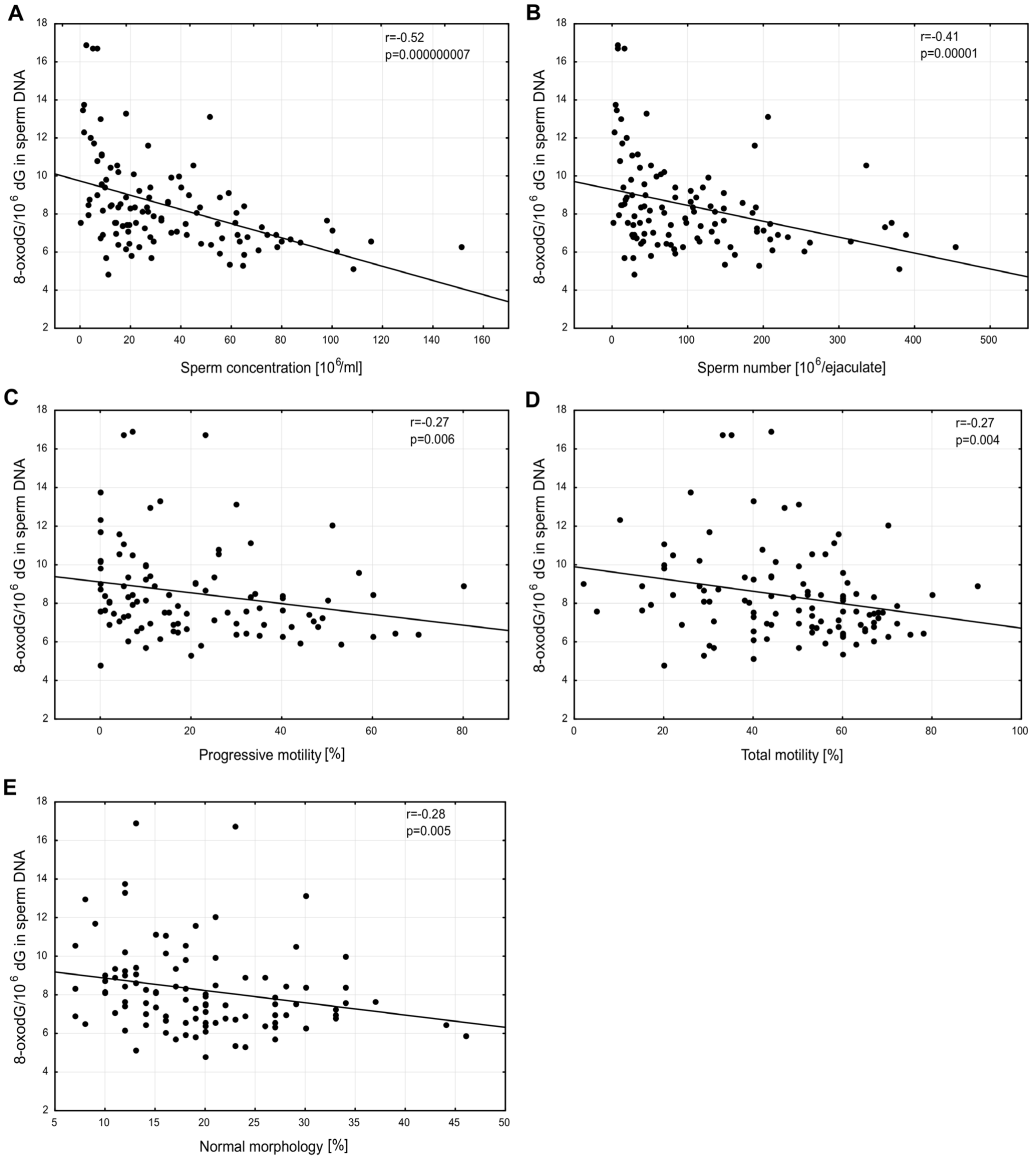
Relationship between 8-oxodG level in sperm DNA and various semen parameters: A, sperm concentration; B, total sperm number; C, progressive motility; D, total motility; E, normal forms of spermatozoa.

For the whole study population a relationship between 8-oxodG levels in sperm DNA and various seminal parameters was analyzed. A highly significant inverse correlation was observed between 8-oxodG level in sperm DNA and sperm concentration (*r* = −0.52; p = 0.000000007) (Figure 2A), for the whole study population. A similar inverse correlation was found both for the control group (*r* = −0.62; p = 0.00017) and for the infertile subjects (*r* = −0.46; p = 0.000024). For the whole study population there was an inverse correlation between 8-oxodG level and sperm number per ejaculate (*r* = −0.41; p = 0.00001) (Figure 2B). A weak inverse correlation was also found between 8-oxodG level and both progressive motility (*r* = −0.27; p = 0.006) and total motility (*r* = −0.27; p = 0.004) as well as between 8-oxodG level in sperm DNA and normal forms of spermatozoa (*r* = −0.28; p = 0.005) (Figure 2C, D, E). No correlation was observed between 8-oxodG levels in leukocyte DNA and semen quality.

**Figure 3:**
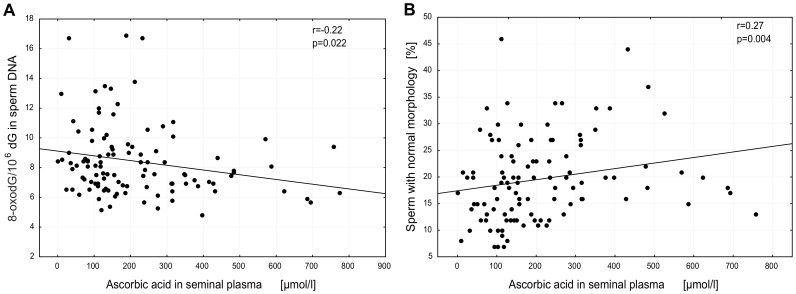
Relationship between seminal plasma ascorbic acid concentration versus: A, level of 8-oxodG in sperm DNA; B, percentage of sperm with normal morphology.

Concentration of several types of antioxidants, including retinol, α-tocopherol, ascorbic acid and uric acid in blood plasma and ascorbic acid and uric acid in seminal plasma were also analyzed. There were no significant differences in blood and seminal antioxidant concentrations between subjects with normozoospermia and abnormal semen parameters (Table 2). A significant albeit weak inverse correlation was observed between ascorbic acid concentration in seminal plasma and level of 8-oxodG in sperm DNA (*r* = −0.22; p = 0.02; Figure 3A). A significant correlation was also found between ascorbic acid concentration in seminal plasma and percentage of spermatozoa with normal morphology (*r* = 0.27; p = 0.004) (Figure 3B).

**Figure 4:**
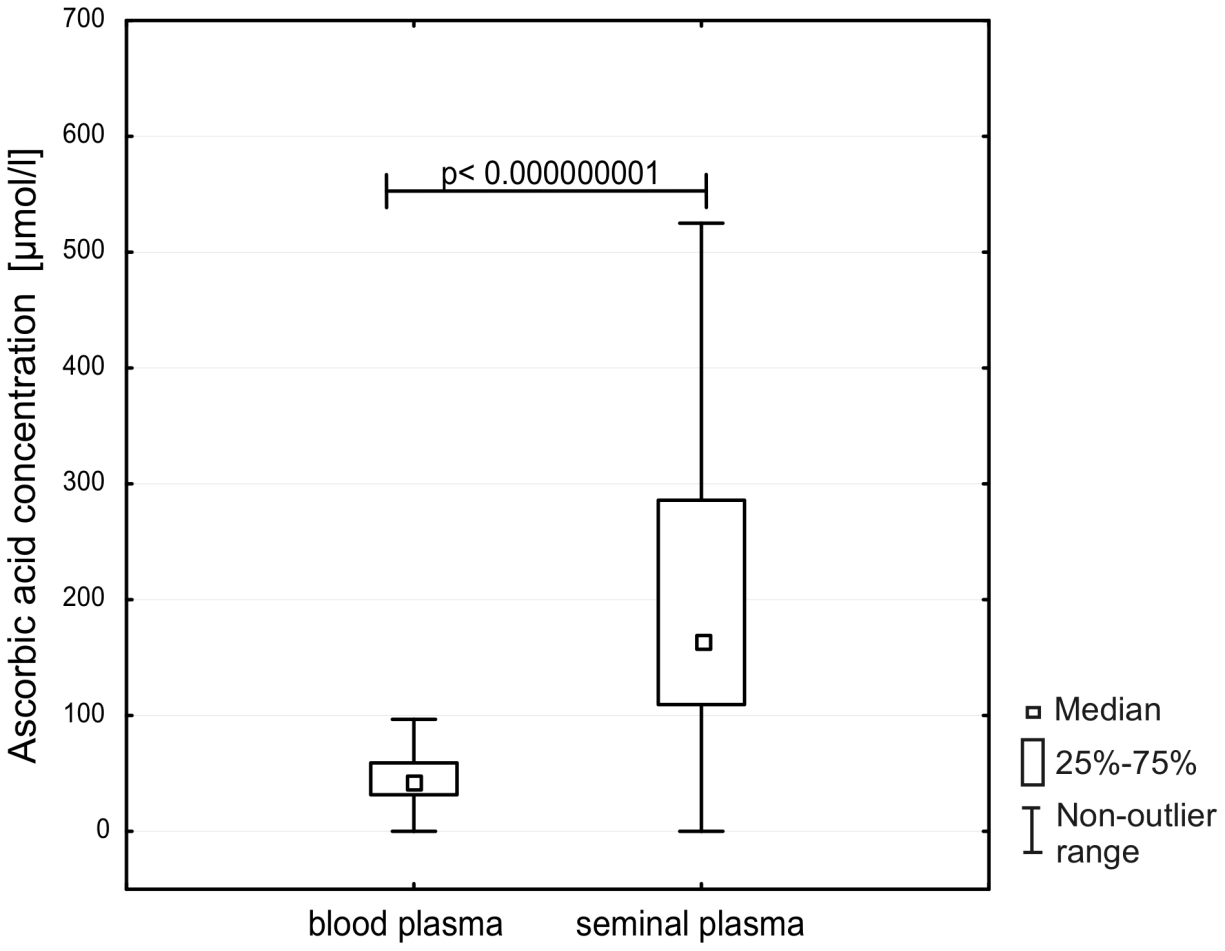
Ascorbic acid concentration in blood plasma and seminal plasma in samples from all studied subjects.

A several-fold higher level of ascorbic acid concentration was observed in seminal plasma compared with blood plasma, the median values being respectively 164.07 (109.41–285.89) versus 43.52 (31.56–59.12); (p<0.000000001) (Figure 4).

**Figure 5:**
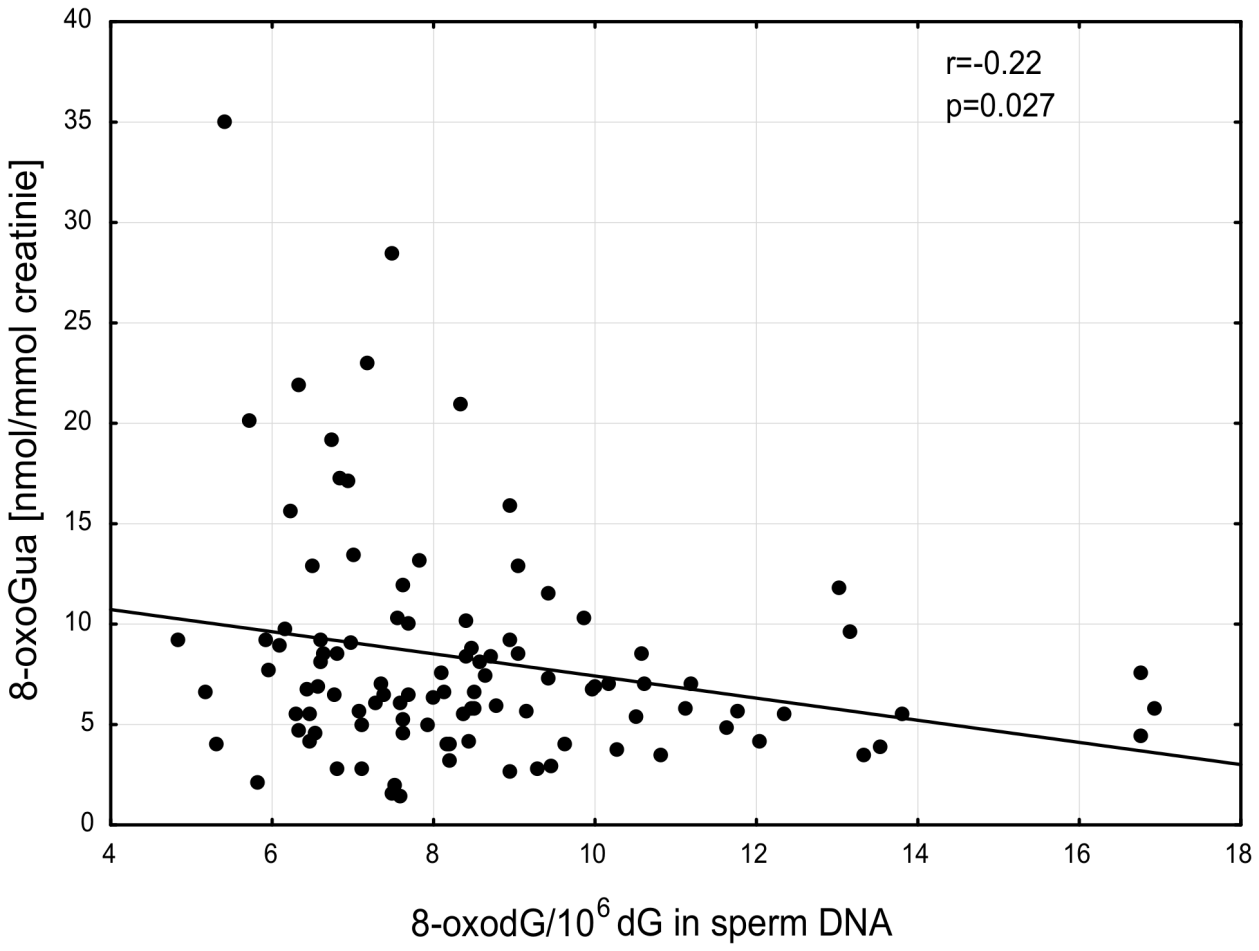
Relationship between urinary excretion 8-oxoGua and the level of 8-oxodG in sperm DNA in all studied subjects.

No significant differences were found in urinary excretion of 8-oxodG in healthy donors versus patients with abnormal semen parameters, whereas a weak inverse correlation was found between 8-oxoGua in urine and 8-oxodG levels in sperm DNA (*r* = −0.22; p = 0.027) (Figure 5).

**Figure 6:**
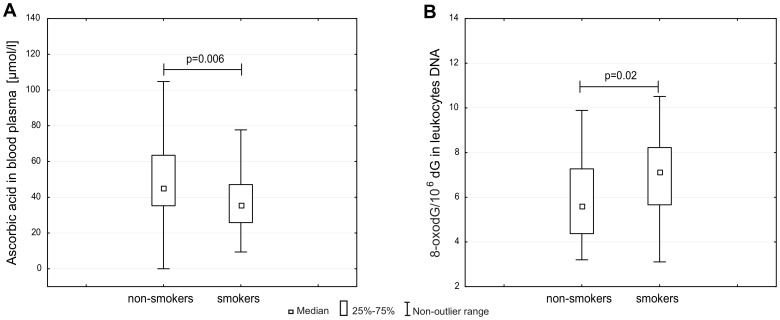
The effect of cigarette smoking on: A, ascorbic acid concentration in blood plasma; B, level of 8-oxodG in leukocyte DNA.

No significant differences were observed for the seminal parameters between smokers and non-smokers. Compared with non-smokers, smoking subjects had decreased levels of vitamin C in blood plasma (median values 45.43 versus 35.88; p = 0.006) (Figure 6A). Significantly higher levels of 8-oxodG in leukocyte DNA were seen in smokers compared with non-smokers (median values 7.14 versus 5.62; p = 0.02) (Figure 6B).

## Discussion

The gold standard to predict male reproductive potential is the definition of sperm quality (concentration, motility, morphology). However, recently it has been recognized that the male factor responsible for fertility as well as correct embryo development depends, at least in part, on the biochemical features of sperm DNA, namely on the level of DNA damage [13]–[15]. One of the main causes of sperm DNA damage/modification is oxidative stress which, in turn, may be directly linked with oxidatively modification of DNA bases.

There have been several studies concerning oxidatively damaged DNA (expressed as 8-oxodG level) in human sperm with an average level in the range 1.0–10.0 per 10^5^ unmodified guanine nucleotides [16]. Some of these data indicated weak inverse correlation of 8-oxodG level with total sperm count and concentration [17]–[21]. There is controversy concerning the relationship between sperm motility or morphology and oxidatively damaged DNA with some reports claiming inverse correlation [19], [22] and a lack of such association in other works [17], [20], [23]. Similar ambiguity was found in the case of smoking status influence on oxidatively damaged sperm DNA [20], [24], [25].

Our data confirm that 8-oxodG level in sperm DNA is linked with male infertility since infertile patients (every group with a certain type of abnormal sperm) contained a highly statistically significant elevated level of the modification than the control group. We have also determined the correlation of 8-oxodG level with seminal parameters and found the highest association between the level of 8-oxodG and sperm concentration (*r* = −0.52, p = 0.000000007). This in turn corroborates the hypothesis which claims that oxidative stress/DNA damage is the major factor involved in apoptosis-like response of male germ line cells [5], [26] which results in errors in spermiogenesis and decreased sperm production [27], [28].

To our best knowledge the present data demonstrates for the first time statistically significant correlations (with p value in the range from 0.000000007 to 0.006) of 8-oxodG level in sperm DNA with every parameter which describes sperm quality: concentration, motility and morphology (Figure 2). Seemingly, a several-times-lower level of modification in DNA detected in our sperm probes than in other works [18]–[20], [25] allowed for more precise analyses. The values presented in this study are in good agreement with those recommended by ESCODD [29], [30].

In agreement with the Loft et al. study [20] we found no difference of 8-oxodG level in sperm DNA between smokers and non-smokers although, in agreement with our previous study [31] we have found a statistically higher level of 8-oxodG in the case of leukocytes DNA of smokers. The aforementioned difference was associated with a significant decrease of ascorbic acid concentration in smokers’ blood (Figure 6). Once again no differences between smokers and non-smokers in ascorbic acid concentration in seminal plasma was found (data not shown). This finding is another argument which underlines the significant protective role of ascorbic acid of sperm plasma against oxidative damage to DNA (see below). The seemingly exceptionally high level of ascorbic acid in sperm plasma constitutes a good buffer to protect sperm DNA against ROS attack generated by smoking.

During the terminal differentiation of spermatozoa chromatin becomes remodeled into an extremely small space which in turn should protect the DNA of these cells against oxidative stress. However, the most striking finding of our study is the elevated level of 8-oxodG in spermatozoa when compared with leukocytes. This difference is highly statistically significant when the whole study population is taken into account (p = 0.00000002). Moreover, the same kind of difference was also found in every study group including the control one (normozoospermia). Although previous reports claim that the level of 8-oxodG in sperm DNA corresponds to other cells in the body [16], [20] no direct comparison was presented. Our data show, for the first time, that 8-oxodG level in sperm DNA is about 25% higher than that in leukocytes DNA. It is noteworthy that both kinds of cell were taken from the same subject. Since leukocytes are regarded as surrogate tissue and 8-oxodG level in these cells is similar to other tissues of the organism [32], [33] the obvious question is what is responsible for the higher level of this potentially harmful/mutagenic modification in spermatozoa? There may be several factors involved in this phenomenon: i/the large number of germ cell divisions (for review see [34]); ii/partial loss of antioxidant protection due to limited volume of cytoplasm [5]; iii/partial inactivation of DNA repair processes and iv/the defective remodeling of chromatin during the final stages of spermatogenesis which can render DNA vulnerable to ROS attack [5]. Although the reasons for the high level of 8-oxodG in spermatozoa is undoubtedly complex the most likely involve oxidative stress and decreased activity of enzymes involved in DNA repair.

Taking into account the whole organism there are large individual differences with respect to coping with oxidative stress/DNA damage reflected in substantial differences in levels of antioxidants, urinary and cellular 8-oxodG level [31], [35]. This variability may reflect individual differences in the metabolism of different oxidants and DNA repair capacity and, at least in part, genetic background [36], [37].

Since the genetic constitution of the offspring depends on the integrity of both sperm and oocyte, there is no doubt that a high level of oxidative damage to DNA, like that detected in infertile men, may lead to zygote arrest or abortion, congenital malformation or disease development including cancer in the offspring. Interestingly, the level of 8-oxodG in sperm DNA of healthy, normozoospermic men is about 20–30% higher than that in control (Table 2, Figure 1C). An intriguing question is why the male organism can tolerate a large amount of this kind of damage despite its potentially adverse effect on fertility and the offspring genetic make-up. An interesting recent hypothesis which offers a kind of answer to this question has been put forward. In a paper published in BioEssay the authors proposed that females are able to distinguish sperm with oxidatively damaged DNA by means of oxidative-dependent signals and select individuals (or rather their sperm) with better DNA quality to fertilize their oocyte [34]. Moreover, based on experimental evidence the authors proposed that the female reproductive tract has certain barriers to filter out oxidatively damaged sperm before reaching the ovum [34]. Intriguingly, our data clearly demonstrated that 8-oxodG in sperm DNA of fertile men (from the control group) is also characterized by a distinct rise in the level when compared with their leukocytes DNA (Figure 1B). This, in turn may suggest that the presence of 8-oxodG in sperm DNA (albeit in lower amount and in restricted chromatin regions) may have a physiological-regulatory function. As a matter of fact it has recently been found that 8-oxodG generation may regulate transcriptional activation [38]–[40]. Similarly, a certain amount of 8-oxodG in sperm DNA may serve as an epigenetic mark which can enable certain genes to be expressed immediately after fertilization.

As mentioned above one of the reasons for the elevated level of 8-oxodG in spermatozoa may be reduced antioxidant capacity due to the limited volume of sperm cytoplasm. To compensate for this loss human seminal plasma contains low molecular weight antioxidants with exceptionally high concentration of ascorbic acid [23], [41], [42]. In good agreement with previous results we found that ascorbic acid concentration was several-fold higher in seminal plasma than in blood (Figure 4). There have been conflicting reports concerning a possible link between ascorbic acid concentration and sperm quality with some studies demonstrating significant differences in this concentration between fertile and infertile men while another reported absence of such differences [41], [43]–[47]. Although we found no differences in ascorbic acid concentration among study groups (Table 2), our results show statistically significant correlation between ascorbic acid concentration in seminal plasma and spermatozoa quality (Figure 3B). Moreover, we found a similar relationship between ascorbic acid concentration in seminal plasma and 8-oxodG level in spermatozoa (Figure 3A). No such association was found for the other analyzed antioxidants, which in turn suggests that ascorbic acid is the main low molecular weight antioxidant to protect spermatozoa DNA integrity (see also above). However, we should admit that the presented correlation is rather weak (in addition to that presented in Figure 5; see below). One of the reasons may be the dynamic flux between the formation and removal of 8-oxoGua by OGG1which in turn may leave abasic sites in place of the adducts.

Another of our findings demonstrated that 8-oxodG level in sperm DNA was inversely correlated with urinary excretion of 8-oxoGua (Figure 5). Excretion of 8-oxoGua into urine represents the average rate of oxidative stress/DNA damage in the whole body. The excretion rate combined with measurement of 8-oxodG in cellular DNA may be helpful to study the question of rate of repair versus rate of damage. Indeed, a number of literature reports and our data indicate that the base excision repair (BER), namely human 8-oxoguanine DNA glycosylase (hOGG1) which removes 8-oxoGua from cellular DNA is responsible for its presence in urine (for a discussion see [8]). Since oxidative stress represented by antioxidant status is similar in different subject groups it is possible that the observed decrease of urinary 8-oxoGua together with simultaneous increase of the level in sperm DNA may reflect a decrease in the activity of hOGG1 which may be genetically determined [48], [49]. In this context it is noteworthy that recently the Aitken group demonstrated the presence of OGG1 in spermatozoa [50]. However, OGG1 mediated BER pathway responsible for 8-oxodG removal is compromised in spermatozoa since the downstream component of BER pathway, namely AP endonuclease (APE1), is absent in these cells [50]. Since it was shown that human APE1 enhances the activity of OGG1 to remove 8-oxodG [51] it is possible that DNA integrity of spermatozoa may be exceptionally dependent on OGG1 and the decrease in its activity may contribute to abnormal spermatozoa and male infertility.

Collectively, the results of our study demonstrate that 8-oxodG level highly statistically significantly correlated with every parameter which describes sperm quality (sperm count, motility and morphology). Moreover, the data indicate highly statistically significant elevated level of 8-oxodG in sperm DNA compared with DNA of surrogate tissue (leukocytes) in infertile men as well as in the healthy control group. Since 8-oxodG level in sperm DNA is inversely correlated with urinary excretion rate of 8-oxoGua, which is the product of OGG1 activity, we hypothesize that the integrity of spermatozoa DNA may be highly dependent on OGG1 activity. We found no relationship between the whole body oxidative stress and that of sperm plasma which suggests that redox status of semen may be rather independent of this characteristic for other tissues.
